# An Adaptive Feature Selection Algorithm for Fuzzy Clustering Image Segmentation Based on Embedded Neighbourhood Information Constraints

**DOI:** 10.3390/s20133722

**Published:** 2020-07-03

**Authors:** Hang Ren, Taotao Hu

**Affiliations:** 1 Changchun Institute of Optics, Fine Mechanics and Physics, Chinese Academy of Sciences, Changchun 130033, China; renhang10@163.com; 2 Key Laboratory of Airborne Optical Imaging and Measurement, Changchun Institute of Optics, Fine Mechanics and Physics, Chinese Academy of Sciences, Changchun 130033, China; 3 School of Physics, Northeast Normal University, Changchun 130024, China

**Keywords:** embedded neighbourhood information, Gaussian mixture model, fuzzy clustering, image segmentation

## Abstract

This paper addresses the lack of robustness of feature selection algorithms for fuzzy clustering segmentation with the Gaussian mixture model. Assuming that the neighbourhood pixels and the centre pixels obey the same distribution, a Markov method is introduced to construct the prior probability distribution and achieve the membership degree regularisation constraint for clustering sample points. Then, a noise smoothing factor is introduced to optimise the prior probability constraint. Second, a power index is constructed by combining the classification membership degree and prior probability since the Kullback–Leibler (KL) divergence of the noise smoothing factor is used to supervise the prior probability; this probability is embedded into Fuzzy Superpixels Fuzzy C-means (FSFCM) as a regular factor. This paper proposes a fuzzy clustering image segmentation algorithm based on an adaptive feature selection Gaussian mixture model with neighbourhood information constraints. To verify the segmentation performance and anti-noise robustness of the improved algorithm, the fuzzy C-means clustering algorithm Fuzzy C-means (FCM), FSFCM, Spatially Variant Finite Mixture Model (SVFMM), EGFMM, extended Gaussian mixture model (EGMM), adaptive feature selection robust fuzzy clustering segmentation algorithm (AFSFCM), fast and robust spatially constrained Gaussian mixture model (GMM) for image segmentation (FRSCGMM), and improve method are used to segment grey images containing Gaussian noise, salt-and-pepper noise, multiplicative noise and mixed noise. The peak signal-to-noise ratio (PSNR) and the error rate (MCR) are used as the theoretical basis for assessing the segmentation results. The improved algorithm indicators proposed in this paper are optimised. The improved algorithm yields increases of 0.1272–12.9803 dB, 1.5501–13.4396 dB, 1.9113–11.2613 dB and 1.0233–10.2804 dB over the other methods, and the Misclassification rate (MSR) decreases by 0.32–37.32%, 5.02–41.05%, 0.3–21.79% and 0.9–30.95% compared to that with the other algorithms. It is verified that the segmentation results of the improved algorithm have good regional consistency and strong anti-noise robustness, and they meet the needs of noisy image segmentation.

## 1. Introduction

Scientific research shows that 70–80% of information in daily life is obtained through the human visual system, and images are an important medium for humans to understand the world and perceive things through. Therefore, images play a very important role in people’s daily lives. For an image, people are generally only interested in some of the content. It is usually necessary to extract the region of interest in an image from the image so that people can best observe this area; then, follow-up processes can be implemented. In short, image segmentation is a process of region division for a given image and the extraction of the target region of interest.

### 1.1. Development Status of Clustering Image Segmentation Algorithms

Among the many region segmentation algorithms, cluster segmentation, as a typical unsupervised segmentation method, has attracted the attention of many scholars and has been widely used and studied in many fields.

Clustering algorithms can be divided into hard partitioning clustering algorithms and soft partitioning clustering algorithms [1,2]. First, an image can be segmented using a hard partition clustering algorithm. The principle is that the image is similarly partitioned based on pixel values of factors such as the greyscale, colour, texture, etc.; then, the optimal solution or partition is obtained by minimising the objective function, including by methods such as the H-means algorithm, global K-means algorithm, K-means algorithm and others. Among them, K-means clustering is a fast segmentation clustering algorithm with a clear structure and good usability, but in the process of optimising the segmentation process, it can also easily fall to a local minimum [3,4].

A soft partitioning clustering algorithm uses the pixel attributes or probability to indirectly divide similar pixels [5], and the optimal decomposition is explored in the process of minimising the objective function or maximising the likelihood function of the parameters, as reported by Dunn in 1947. In 2001, the fuzzy C-means clustering algorithm (FCM) was proposed. Then, in 1981 [6], Bezdek verified metric theory by comparing mean clustering and fuzzy mean clustering. The convergence of the fuzzy mean clustering algorithm was validated, fuzzy clustering theory was established, and the development of the fuzzy clustering algorithm was promoted, thus making the fuzzy mean clustering algorithm an important branch of fuzzy theory. Introducing this theory into the clustering algorithm improved the adaptability of the algorithm, and this approach is currently widely used [7,8].

An image segmentation method based on subspace clustering was proposed in [9,10]. They defined the search strategies and evaluation criteria to filter out the features that are effective for clustering; then, they clustered the original data set in different subspaces and reduced the storage and calculation requirements.

The existing supervised feature selection methods [11,12] can reduce the problem dimensions, but the operating efficiency decreases. Therefore, adaptive feature selection is often used to achieve clustering segmentation. A similarity measurement method for high-dimensional data was proposed in the literature considering the correlations among high-dimensional spatial features, and this approach effectively avoids “dimensional disasters”. The impact of high-dimensional data has been addressed, but there is still a lack of theoretical guidance on how to select the criteria for similarity measures. To avoid a combined search [13], which is only suitable for unsupervised learning, the concept of feature saliency was proposed; the related approach considers the influence of different features on the clustering effect and uses a Gaussian mixture model for clustering analysis to improve the algorithm’s performance.

An iterative, structured, low-rank optimisation method for multiview spectral clustering was proposed [14]. Unlike existing methods, this method can effectively encode the local data manifold structure from each view-dependent feature space and achieve multiview agreement in an iterative fashion while preserving the flexible nonlinear manifold structure of all views. A clustered low-rank representation of structured matrix factorisation for multiview spectral clustering was proposed [15]. Unlike the existing methods, in this approach, an iterative strategy for intuitively achieving multiview spectral clustering agreement by minimising the between-view divergence in terms of the factorised latent data cluster representation for each view is implemented.

### 1.2. Feature Selection Algorithm with a Fuzzy Gaussian Mixture Model and the Related Limitations

The FCM uses the squared Euclidean distance to characterise the difference between the samples and the clustering centre, but this approach is only suitable for clustering data samples that have different sizes, variances and classes.

Therefore, a fuzzy Gaussian mixture mode (FGMMS) is proposed [16]. The Gaussian mixture model is used as a distance measure to replace the Euclidean distance in the fuzzy C-means algorithm. The distance is replaced by the results of the Gaussian mixture model, which can fit multi-peak data more accurately than the traditional approach and better segment noise-free, complex images. The traditional fuzzy C-means clustering analysis treats the different features of samples as clusters and ignores the important effects of key features on the clustering results; therefore, the clustering results are far from the real classification results. According to feature selection theory, we can use the concept of feature saliency, assume that the sample saliency obeys a probability distribution, and conduct cluster analysis using the Gaussian mixture model.

Online feature selection based on fuzzy clustering and its applications was proposed (OFSBFCM) [17,18]. This algorithm introduces feature selection using the fuzzy Gaussian mixture model and obtains a new fuzzy clustering method. A fuzzy C-means clustering method that combines feature selection with the Gaussian mixture model using Kullback–Leibler (KL) divergence was proposed as a type of Gaussian mixture model (GMM) with Markov random fields; this approach has become a research hotspot in the field of image segmentation [19,20]. When a GMM is directly applied for image segmentation, it is easily disturbed by noise. Many scholars have introduced the spatial information from neighbouring pixels into the prior probability distribution of the GMM to reduce the sensitivity of the algorithm to noise.

A spatial constraint method for the Gaussian mixture model was proposed [21] based on the extended Gaussian mixture model (EGMM), which is used to construct the neighbourhood information weight function with the prior probability to constrain the pixel space and improve the noise robustness of the GMM.

A fast and robust spatially constrained GMM was proposed [20] (fast and robust spatially constrained GMM for image segmentation, FRSCGMM), but this algorithm does not consider the impact of different features on clustering, and it still has limitations in high-noise-region segmentation [22].

Since the fuzzy local information C-means (FLICM) segmentation algorithm cannot consider the effects of different features on the clustering segmentation results, a local fuzzy clustering segmentation algorithm based on a feature selection GMM is proposed. First, the membership degree is introduced into the local constraint information of the FLICM algorithm. Considering the influence of features on clustering, the feature saliency is then introduced into the objective function of the algorithm. Finally, the neighbourhood weighting function is constructed using the classification membership degree, and the membership degree is processed to obtain the feature-based membership. The local fuzzy clustering algorithm is selected. The improved algorithm was compared with the existing robust clustering segmentation algorithms in a clustering segmentation test with noisy images. The segmentation results were objectively compared based on the peak signal-to-noise ratio (PSNR) and error rate, which verified the effectiveness and practicability of the proposed algorithm [23].

When the Feature selection Gaussian mixture model (FSGMM) is applied for greyscale image segmentation, it has difficulties suppressing the influence of noise on the image segmentation result. Notably, the algorithm does not consider the fact that any pixel in the image is closely related to its neighbouring pixels [24,25].

The problems of the fuzzy clustering algorithm based on the feature selection GMM are as follows:

1. The parameters need to be adjusted to increase the run time of the algorithm [26,27].
2. As in the FCM algorithm, the FSGMM only clusters single pixels without considering the influence of the spatial neighbourhood pixels on each central pixel. For images with different types of noise interference, the algorithm does not have good noise robustness [28,29].
3. The fuzzy clustering algorithm in the FSGMM does not take the neighbourhood space information into account, so it is difficult to limit the influence of noise on image segmentation, resulting in noise sensitivity [30].

### 1.3. Main Contributions of This Paper

In this paper, the FSGMM fuzzy clustering algorithm does not consider the neighbourhood space information; therefore, it is difficult to limit the influence of noise on image segmentation, resulting in a noise sensitivity problem. In this paper, an adaptive feature selection algorithm based on embedded neighbourhood information constraints is proposed. The definition of the prior probability of the Markov random field is established. The Markov random field is constructed by creating a new spatial neighbourhood information function, which is embedded in the FSGMM fuzzy clustering algorithm; then, the noise smoothing factor is introduced to further improve the anti-noise robustness of the algorithm. Through the improved algorithm, remote sensing images with noise and simulated images are divided into two categories, and multiple experiments are conducted to analyse the performance of the algorithm. Compared with several typical clustering algorithms, the improved algorithm has good regional consistency and strong noise robustness, and it meets the needs of noisy image segmentation. The peak signal-to-noise ratio (PSNR) and the error rate (MCR) are used as the theoretical basis for the segmentation results. For the anti-Gaussian noise, the PSNRs of the algorithm in this paper are 16.0013, 25.5661, 16.4271, 13.5148 and 27.2172, and the MCRs are 10.12, 1.78, 1.96, 3.87 and 1.96. For salt-and-pepper noise, the PSNRs of the algorithm in this paper are 12.9512, 17.2612, 22.7521, 21.7545 and 23.7612, and the MCRs are 13.17, 6.12, 2.51, 3.14 and 4.23. For multiplicative noise, the PSNRs of the algorithm in this paper are 22.4898, 15.2874, 23.5412 and 13.7645, and the MCRs are 2.18, 2.58, 2.12 and 1.45. For mixed noise, the PSNRs of the algorithm in this paper are 15.4321. 16.7235, 18.5821, 14.2123 and 13.2356, and the MCRs are 11.21, 5.21, 3.61, 2.31 and 3.21. Compared with other algorithms, the proposed algorithm yields increases of 0.1272–12.9803 dB, 1.5501–13.4396 dB, 1.9113–11.2613 dB and 1.0233–10.2804 dB, and the MSR decreases by 0.32–37.32%, 5.02–41.05%, 0.3–21.79% and 0.9–30.95%. Notably, the improved algorithm yields the best indicators in all cases.

## 2. Algorithm Analysis

In the process of clustering segmentation, considering the important influence of the key features of samples of the clustering results, clustering analyses of feature selection based on the concept of weighting have been widely studied and performed. In addition, to improve the anti-noise robustness of this kind of algorithm, GMMs have been fused with Markov random fields—a research hotspot in the field of image segmentation.

### 2.1. FSGMM Fuzzy Clustering Algorithm

When the FSGMM clustering algorithm is applied in greyscale image segmentation, it has difficulties suppressing the influence of noise on the image segmentation result. The fundamental reason is that the algorithm does not take into account any pixels in the image that are closely related to the surrounding pixels [31]. Thus, in the extended GMM, we assume that the neighbouring pixels and the central pixel follow the same distribution; that is, they have the same distribution parameters (mean and covariance). Then, the sum of the probability that the neighbouring pixels are of various types is weighted by the exponential power. To construct a Markov random field, the spatial neighbourhood information function is expressed as follows [32,33,34].

(1) $$\pi_{ij}=\sum_{l=1}^{D}\frac{h_{jl}}{\sum_{k=1}^{C}h_{kl}}$$

(2) $$\mathrm{where}\;h_{jl}=\{\sum_{t\in N_{i}}\Phi_{ij}{\left(\Phi (x_{il}|\mu_{jl},\sigma_{jl}^{2})\right\}}^{2}$$

(3) $$\Phi_{jl}(x_{il}|\mu_{jl},\sigma_{jl}^{2})=\frac{1}{\sqrt{2\pi \sigma_{jl}^{2}}}\exp\{-\frac{{\left(x_{il}-\mu_{jl}\right)}^{2}}{2\sigma_{jl}^{2}}\}$$

The neighbourhood information function is used as the a priori probability in the FSGMM fuzzy clustering algorithm, and an adaptive feature selection robust fuzzy clustering segmentation algorithm (AFSFCM) is proposed. The objective function of the algorithm is

(4) $$\min L(\Theta )=\sum_{i=1}^{N}\sum_{j=1}^{C}z_{ij}d_{ij}+\lambda \sum_{i=1}^{N}\sum_{j=1}^{C}(z_{ij}\log\frac{z_{ij}}{\pi_{ij}})+\gamma \sum_{i=1}^{N}\sum_{j=1}^{C}\sum_{l=1}^{D}z_{ij}(s_{ijl}\log\frac{s_{ijl}}{\rho_{l}}+(1-s_{ijl})\log\frac{1-s_{ijl}}{1-\rho_{l}})$$

where $\pi_{ij}$ is the prior probability that pixel $x_{i}$ belongs to category j, and $\Phi_{ij}(x_{tl}|\mu_{jl,}\sigma_{jl}^{2})$ represents the probability that the l-dimensional feature attribute value corresponding to the neighbouring pixel $x_{t}$ of the centre pixel $x_{i}$ belongs to category j.

$x_{tl}(t\in N_{i})$ represents the l-dimensional feature attribute value corresponding to pixel $x_{i}$ in the square neighbourhood window, where the centre pixel is $x_{i}$; $h_{jl}$ represents the probability that the l-dimensional feature attribute value corresponding to the neighbourhood pixel $x_{t}$ of the centre pixel $x_{i}$ belongs to class j; α represents the weight; and the constant K = 14 is generally used. We need to solve the segmentation model to obtain the $z_{ij},\mu_{jl},s_{ijl,}\sigma_{jl}^{2}$-dependent iteration expression corresponding to the iterative solution to the problem. First, we find the partial derivative of $s_{ijl}$ using Equation (4)

(5) $$\frac{\partial J}{\partial s_{ijl}}=z_{ij}(-\log\Phi (x_{il}|\mu_{jl},\sigma_{jl}^{2})+\log\Phi (x_{il}|\varepsilon_{l},\nu_{l}^{2})+\gamma \log\frac{s_{ijl}}{\rho_{l}}-\gamma \log\frac{1-s_{ijl}}{1-\rho_{l}}$$

Let $\frac{\partial J}{\partial s_{ijl}}=0$. The B iteration expression is

(6) $$s_{ijl}=\frac{\rho_{l}\Phi_{jl}{\left(x_{jl}|\mu_{jl},\sigma_{jl}^{2}\right)}^{1/\gamma }}{\rho_{l}\Phi (x_{il}|\mu_{jl},\sigma_{jl}^{2}+(1-\rho_{l})\Phi {\left(x_{il}|\varepsilon_{l},\nu_{l}^{2}\right)}^{1/\gamma }}$$

Using the Lagrangian multiplier method for solving constrained optimisation problems, we obtain the partial derivative of $z_{jl}$.

(7) $$\frac{\partial }{\partial z_{ij}}[J-\sum_{i=1}^{N}\eta_{i}(\sum_{j=1}^{C}z_{ij}-1)]=0$$

From Equation (7), the iterative membership function can be derived as follows:

(8) $$t_{ij}=-\gamma \sum_{l=1}^{D}(\rho \Phi {\left(x_{il}|\mu_{jl},\sigma_{jl}^{2}\right)}^{1/\gamma }+(1-\rho )\Phi {\left(x_{il}|\varepsilon_{l},\nu_{l}^{2}\right)}^{1/\gamma }$$

The partial derivatives of $\mu_{ij}$ and $\sigma_{jl}^{2}$ give

(9) $$\frac{\partial J}{\partial \mu_{jl}}=\sum_{i=1}^{N}z_{ij}s_{ijl}\sigma_{jl}^{-2}(\mu_{jl}-x_{il})$$

(10) $$\frac{\partial J}{\partial \sigma_{jl}^{2}}=\frac{1}{2}\sum_{i=1}^{N}z_{ij}s_{ijl}(\sigma_{jl}^{-2}-\sigma_{jl}^{-4}{\left(x_{il}-\mu_{jl}\right)}^{2})$$

Let $\frac{\partial J}{\partial \sigma_{jl}^{2}}=0$ and $\frac{\partial J}{\partial \mu_{jl}}=0$. The iteration formulas of $\mu_{jl}$ and $\sigma_{jl}^{2}$ can be obtained as

(11) $$\mu_{jl}=\frac{1}{M_{jl}}\sum_{i=1}^{N}z_{ij}s_{ijl}x_{il}$$

(12) $$\sigma_{jl}^{2}=\frac{1}{M_{jl}}\sum_{i=1}^{N}z_{ijl}s_{ijl}{\left(x_{il}-\mu_{jl}\right)}^{2}$$

(13) $$\frac{\partial J}{\partial \varepsilon_{l}}=\sum_{i=1}^{N}\sum_{j=1}^{N}z_{ij}(1-s_{ijl})\nu_{l}^{-2}(\varepsilon_{l}-x_{il})$$

(14) $$\frac{\partial J}{\partial \nu_{l}^{2}}=\sum_{i=1}^{N}\sum_{j=1}^{C}z_{ij}(1-s_{ijl})(\nu_{jl}^{-2}-\nu_{jl}^{-4}{\left(x_{il}-\varepsilon_{l}\right)}^{2})$$

Let $\frac{\partial J}{\partial \varepsilon_{l}}$ = 0 and $\frac{\partial J}{\partial \nu_{l}^{2}}$ = 0. It can be concluded that the iterative expressions of $\varepsilon_{l}$ and $\nu_{l}^{2}$ are

(15) $$\varepsilon_{l}=\frac{1}{M_{jl}}\sum_{i=1}^{N}\sum_{j=1}^{C}z_{ij}(1-s_{ijl})x_{il}$$

(16) $$\nu_{l}^{2}=\frac{1}{F_{l}}\sum_{i=1}^{N}\sum_{j=1}^{C}z_{ij}(1-s_{ijl}){\left(x_{il}-\varepsilon_{l}\right)}^{2}$$

(17) $$\mathrm{where}\;M_{jl}=\sum_{i=1}^{N}z_{ij}s_{ijl}x_{il}$$

Next, we calculate the partial derivative of $\rho_{l}$ to obtain

(18) $$\frac{\partial }{\partial \rho_{l}}[J-\nu (\sum_{j=1}^{C}\rho_{l}-1)]=0$$

The iterative expression of $\rho_{l}$ is

(19) $$\rho_{l}=\frac{1}{N}\sum_{i=1}^{N}\sum_{j=1}^{C}z_{ij}s_{ijl}$$

This equation is the GMM algorithm for feature selection and image segmentation with the neighbourhood information constraint.

### 2.2. Adaptive FSMM Fuzzy Clustering Image Segmentation Algorithm Based on the Embedded Neighbourhood Information Constraint

To further improve the anti-noise robustness of the algorithm, the noise smoothing factor is embedded in the adaptive feature selection robust fuzzy clustering segmentation algorithm, and a new Gaussian hybrid algorithm for neighbourhood information feature selection is obtained [35,36,37].

(20) $$\begin{array}{l} \min J(\Theta )=\sum_{i=1}^{N}\sum_{j=1}^{C}z_{ij}d_{ij}+\lambda \sum_{i=1}^{N}\sum_{j=1}^{C}(z_{ij}\log\frac{z_{ij}}{\pi_{ij}}+G_{ij}\log\frac{G_{ij}}{\pi_{ij}})\\ +\gamma \sum_{i=1}^{N}\sum_{j=1}^{C}\sum_{l=1}^{D}z_{ij}(s_{ijl}\log\frac{s_{jl}}{\rho_{l}}+(1-s_{ijl})\log\frac{1-s_{ijl}}{1-\rho_{l}}) \end{array}$$

Here, $\pi_{ij}$ means that after adding the noise smoothing factor, each sample $x_{ij}$ corresponds to the a priori probability that the l-dimensional feature belongs to category j. $G_{ij}$ is the noise smoothing factor, which is defined as a power function of the sum of the neighbourhood information weight function (prior probability) $h_{ij}$ and the membership degree $z_{ij}$ (post probability), and the expression is as follows:

(21) $$G_{ij}=\exp(\frac{\beta }{2N_{i}}\sum_{t\in N_{i}}\left[z_{tj}+h_{tj}]\right)$$

In the above formula, the weight coefficient β is used to adjust the influence of the neighbourhood mean on the central pixel and control the ability to smooth noise. In this paper, β=12 is used. $N_{i}$ is the number of pixels in the surrounding neighbourhood, and $N_{i}=25$ is selected. $z_{tj}$ represents pixel $x_{il}$, the neighbouring pixel belongs to the membership degree of the j class, and $h_{ij}$ is the weight function of neighbourhood information. In this paper, the Gaussian distribution of the neighbourhood information is used, and the Gaussian normal weight coefficient $\Phi_{ij}(x_{il}|\mu_{ij},\sigma_{jl}^{2})$ of the neighbourhood information is normalised to obtain a new neighbourhood information weight function. α represents the weight. The test results show that a satisfactory segmentation effect can be obtained when α=14.

The optimal expression for the objective function is solved to obtain the $s_{ijl}$, $z_{ij}$, $\mu_{jl}$ and $\sigma_{jl}^{2}$ corresponding to the iterative solution to the problem. The expressions are the same as those in Equations (6)–(18). The improved algorithm adds a noise smoothing factor, and the improved neighbour domain information weight prior probability $\pi_{ij}$ is obtained. Using the Lagrange multiplier method [38,39,40], the unconstrained objective function optimisation expression is

(22) $$\min L=\sum_{i=1}^{N}\eta_{i}(\sum_{j=1}^{C}\pi_{ij}-1)$$

The above formula is used to find the partial derivative of the prior probability $\pi_{ij}$ and make the partial derivative 0. The solution is

(23) $$\pi_{ij}=\frac{z_{ij}+G_{ij}}{\sum_{k=1}^{C}z_{ik}+G_{ik}}$$

By combining Equations (6)–(23), the specific steps in the improved adaptive feature selection and robust fuzzy clustering segmentation algorithm are described as follows.

1. Initialise the feature attribute weight coefficient ρ=1/D(l=1,……,D) and the prior probability $\pi_{j}=1/C(j=1,\ldots ,C)$ of the given category. Set the regularisation parameter as λ=150 and feature parameter as γ=15.
2. Use FCM clustering. The sample classification class centre vector is $\mu_{j}=(\mu_{j1},\ldots ,\mu_{jD})$, $\sigma_{j}^{2}=(\sigma_{j1}^{2},\ldots ,\sigma_{jD}^{2})$ is a class variance matrix, the sample classification membership is $z_{ij}$, the sample feature mean vector is $\varepsilon =(\varepsilon_{1},\ldots ,\varepsilon_{D})$, and the feature variance matrix is $\nu^{2}=(\nu_{1}^{2},\ldots \nu_{D}^{2})$.
3. Use Equation (19) to update the smoothing factor $G_{ij}$.
4. Use Equation (22) to update the prior probability $\pi_{ij}$.
5. Use Equation (7) to calculate the degree of membership of the characteristic attribute $s_{ijl}$.
6. Use Equation (9) to calculate the classification membership $z_{ij}$.
7. Use Equations (12)–(19) to update the clustering centre vector $\mu_{i}$, class variance matrix $\sigma_{j}^{2}$, feature mean vector ε and feature variance matrix $\nu^{2}$.
8. If the maximum number of iterations is reached or the convergence condition $\{|L{\left(\Theta \right)}^{\left(t+1\right)}-L{\left(\Theta \right)}^{\left(t\right)}|\}<\delta$ is satisfied, stop the iteration; otherwise, return to Step 3, and continue the iteration.

## 3. Experimental Results and Analysis

**Figure 1:**
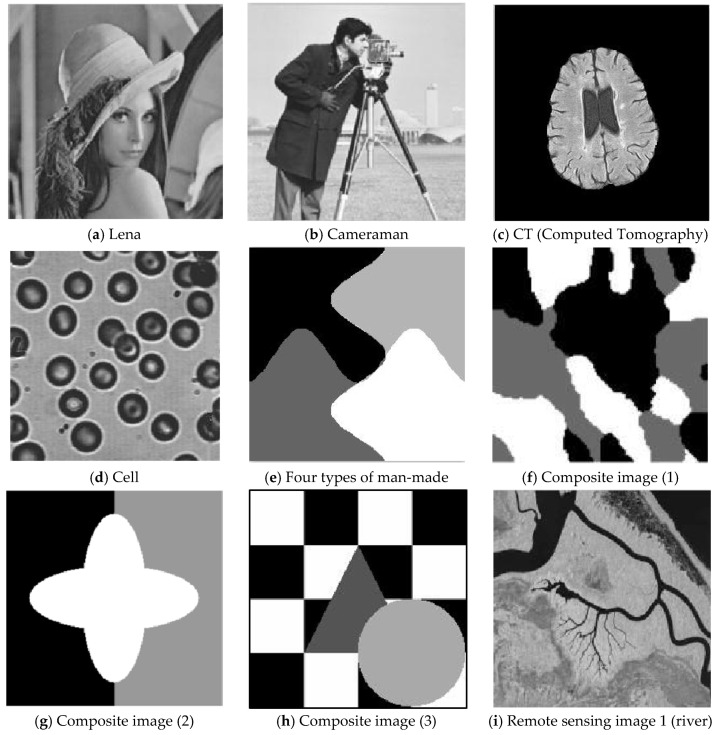

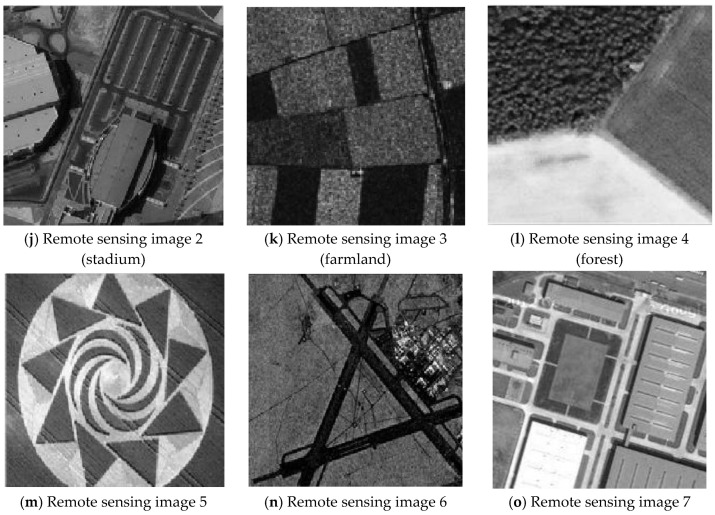
The original image.

To verify the segmentation performance and anti-noise robustness of the improved algorithm, the FSFCM, SVFMM, EGFMM, EGMM, AFSFCM and FRSCGMM were used for comparison. We compared greyscale images with different noise levels, as shown in Figure 1. The peak signal noise was used. The ratio and error rate were used as the theoretical basis for assessing the segmentation result. The testing platform is as follows [41,42,43]: an Intel Core I7 computer with 8 GB of memory, the Windows 10 system, and the MATLAB 2013a programming environment.

Before clustering data sets, the number of clusters C must be given; otherwise, the clustering algorithm will not work. However, there is still no feasible standard for determining the number of clusters, and this value is often selected based on experience. Therefore, the determination of the number of clusters is the main difficulty with the mean clustering method. After many experiments, C was chosen as 2, 3 and 4.

For the regularisation parameter λ in the optimal model of robust fuzzy clustering, the performance and denoising ability of image clustering segmentation are quite different. If the pixel is an isolated noise point, the greater the difference between the pixel and the neighbourhood pixels—that is, the larger the weight—the more reasonable it is to replace the pixel with the mean or median of the neighbourhood; on the contrary, the smaller the weight is, the lesser the influence of the neighbourhood information on the current pixel. For this data set, the regularisation parameter is selected as λ=150.

For a low level of noise, a 3×3 window can be selected for segmentation; for a high level of noise, to maintain a balance between the segmentation accuracy and segmentation time, a 5×5 window is more suitable for segmentation.

As the α control parameter increases, the clustering results are increasingly affected by the spatial pixels; on the contrary, the influence of the neighbourhood pixels is reduced. If this value is too large, the clustering process will rely excessively on the neighbourhood information, and the image details will be blurred. The test results show that α=14 and the segmentation effect is satisfactory.

γ, δ and $T_{\max}$ are selected by the algorithm in this paper, and many experiments were performed to select γ=15, $\delta =10^{-4}$ and $T_{\max}=400$ for the image data set used.

We set the maximum number of iterations in the algorithm to 400; the number of clusters C is selected as 2, 3 and 4; the regularisation parameter is set as λ=150; the characteristic parameter is set as γ=15; the threshold is $\delta =10^{-4}$; and the neighbourhood window size is set to 5×5.

To quantitatively evaluate the anti-noise robustness of different algorithms, the PSNR for traditional image quality evaluation is used. The PSNR is defined as:

(24) $$MSE=\frac{1}{mn}\sum_{i=1}^{N}\sum_{j=1}^{C}{\left\|I_{1}(i,j)-I_{2}(i,j)\right\|}^{2}$$

(25) $$PSNR=10\cdot \log_{10}(\frac{MAX^{2}}{\sqrt{MSE}})$$

where $I_{1}$ represents the segmentation result of the clustering algorithm after adding noise to the image; $I_{2}$ represents the ideal segmentation result of the image without noise; MAX represents the maximum grey level of the image, which is generally 255; and MSE is the mean square deviation of the segmentation results, which reflects the destructive effect of noise on the image segmentation accuracy. The PSNR reflects the anti-noise robustness of the image segmentation result after adding noise based on the ratio of the square of the peak pixel value to the mean square deviation. The value of the PSNR is directly proportional to the strength of the anti-noise performance. With an increase in the PSNR, the anti-noise performance of the algorithm is also enhanced.

Generally, the misclassification rate (MCR, misclassification rate) is used to quantitatively evaluate the performance of a segmentation algorithm, and it is defined as [44,45,46]

(26) $$MCR=[1-(\sum_{j=1}^{C}C_{j}{)}^{-1}.(\sum_{j=1}^{C}A_{j}\cap C_{j})]\times 100\%$$

Here, $A_{j}$ represents the type-j sample point obtained from the image using a certain segmentation algorithm, and $C_{j}$ represents the type-j sample point corresponding to the ideal segmentation result. The smaller the value of the MCR calculation result, the better the segmentation performance of the algorithm [47,48,49].

### 3.1. Image Segmentation Test with Gaussian Noise

**Figure 2:**
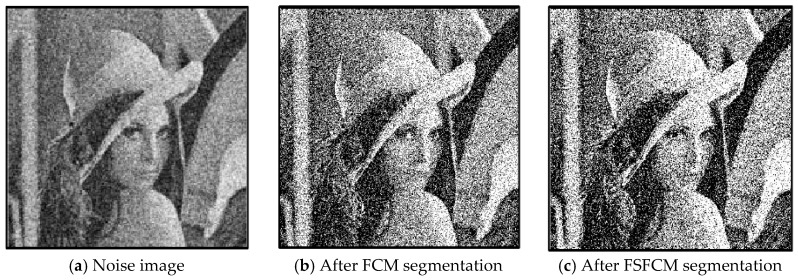

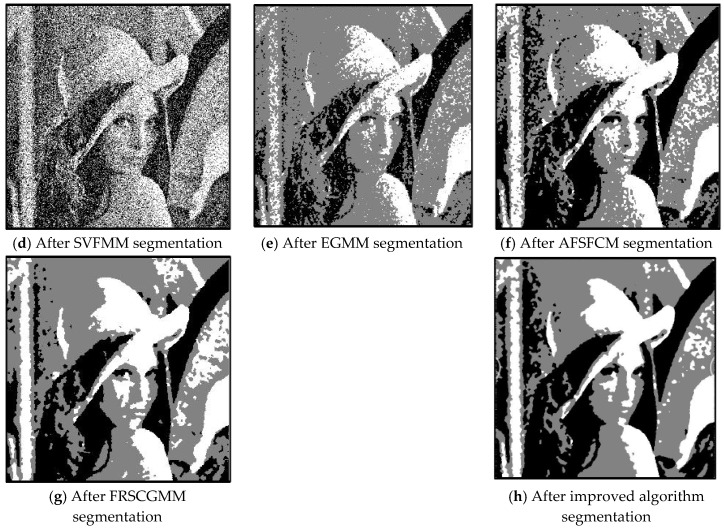
Gaussian noise interfering with the Lena image and the segmentation results.

**Figure 3:**
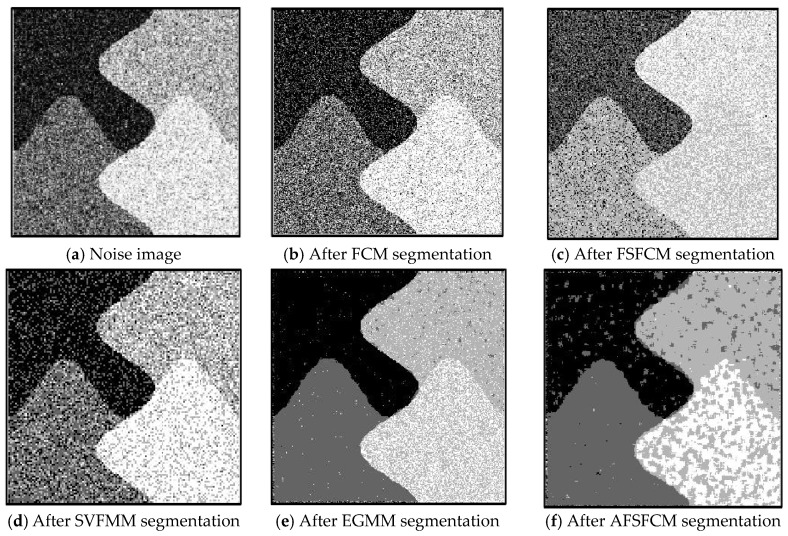

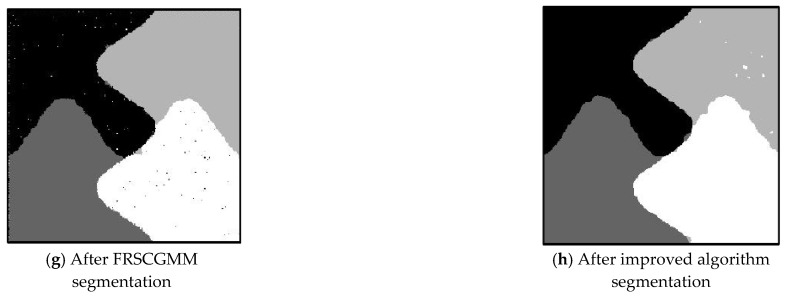
Gaussian noise interfering with the images of the four types of man-made objects and the segmentation results.

**Figure 4:**
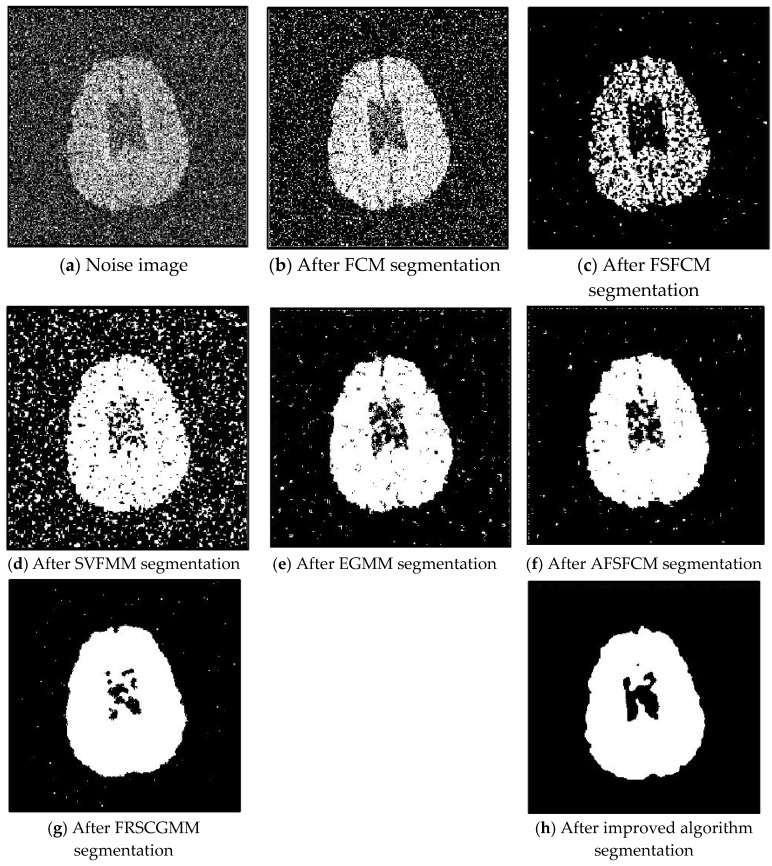
Gaussian noise interferes with brain slice images and the segmentation results.

**Figure 5:**
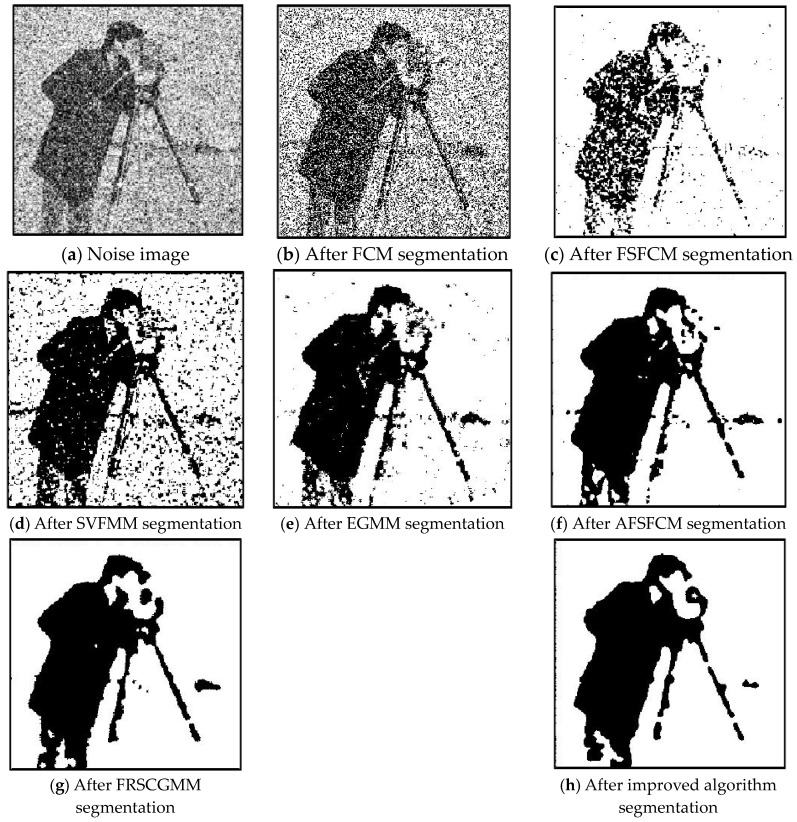
Gaussian noise interfering with the image and the segmentation results for the Cameraman.

**Figure 6:**
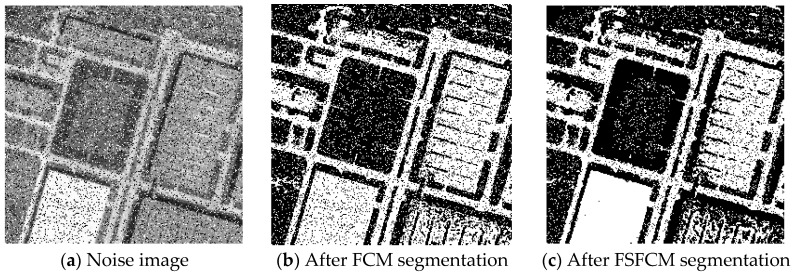

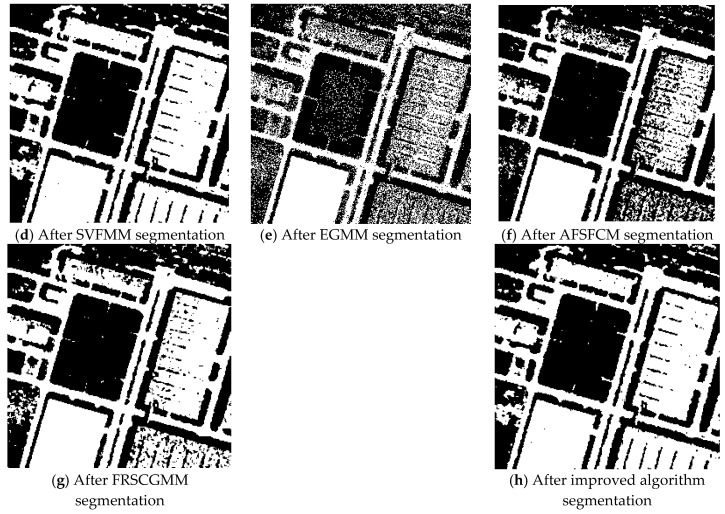
Gaussian noise interfering with the image and the segmentation results for remote sensing image 7.

In the two greyscale images of Lena and the man-made objects, we add Gaussian noise with a mean value of 0 and a mean squared error of 58 and Gaussian noise with a mean value of 0 and a mean squared error of 85. Gaussian noise with a mean value of 0 and a mean square error of 140 is added to the videographer and brain slice images for the segmentation test. We add Gaussian noise with a mean value of 0 and a mean square deviation of 135 to remote sensing image 7. The numbers of clusters are 3, 4, 2 and 2, respectively. The segmentation results are shown in Figure 2, Figure 3, Figure 4, Figure 5 and Figure 6, and the corresponding PSNRs and error rates are shown in Table 1 and Table 2, respectively.

From the anti-Gaussian noise segmentation results in Figure 2, Figure 3, Figure 4, Figure 5 and Figure 6, it can be observed that the FCM, FSFCM and SVGMM algorithms are heavily influenced by Gaussian noise, and the EGMM algorithm has a certain ability to smooth noise. The improved AFSFCM algorithm contains a reduced noise point, and the FRCSGMM algorithm introduces a noise smoothing factor to suppress most of the noise. However, compared with the improved algorithm, the FRSCGMM algorithm still contains more noise points in the segmentation results, and the improved algorithm contains less noise; additionally, the edges are clear. Table 1 shows that the peak signal-to-noise ratio of the improved algorithm is at least 4 dB higher than the signal-to-noise ratio of the FCM algorithm, and the proposed method has a higher PSNR value than the other five algorithms, indicating that the improved algorithm is more resistant to Gaussian noise. Then, from the error rates of the segmentation results shown in Table 2, the MCR of the segmentation result of the improved algorithm is much smaller than the MCRs of the other four algorithms. The PSNR of the improved algorithm is 0.1272–12.9803 dB higher than that of the other algorithms, and the MCR is 0.32–37.32% lower than that of the other algorithms. According to the above analysis, the FRCSGMM and EGMM algorithms have a certain anti-noise robustness, but the improved algorithm achieves better noise resistance and segmentation performance.

### 3.2. Image Segmentation Test for Salt-and-Pepper Noise

**Figure 7:**
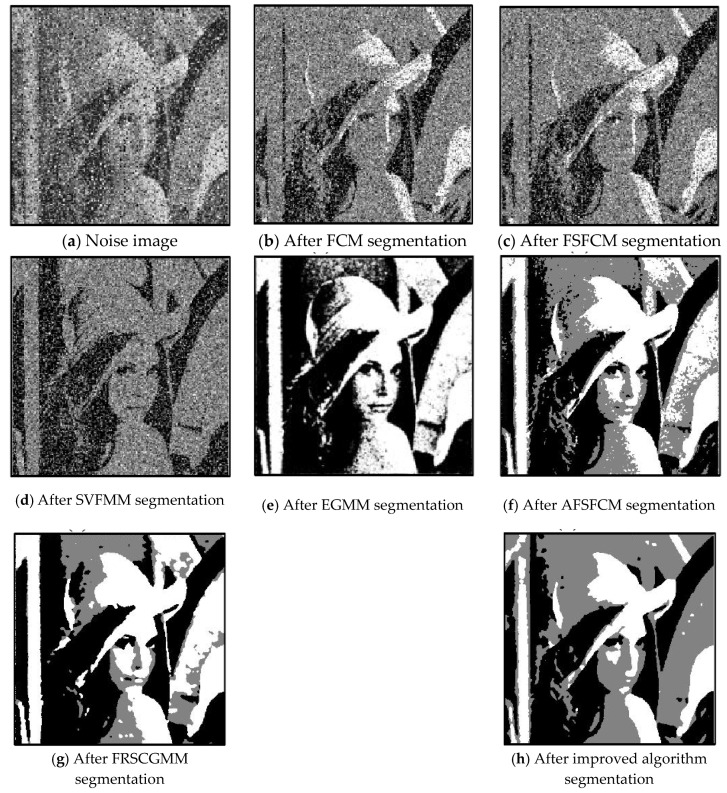
Salt-and-pepper noise interfering with the Lena image and the segmentation results.

**Figure 8:**
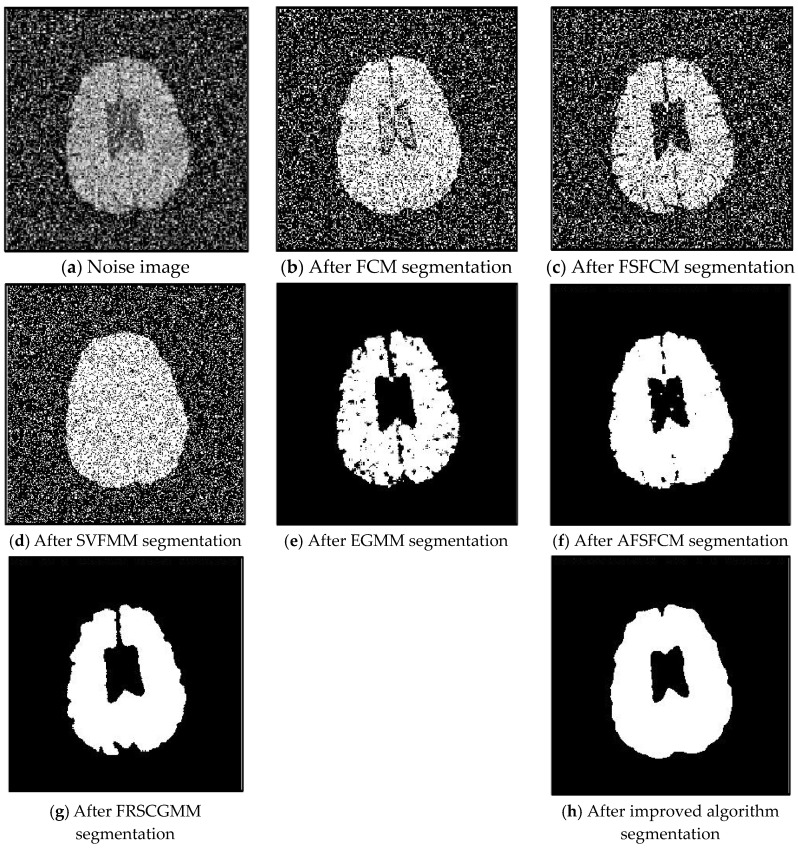
Salt-and-pepper noise interfering with the brain slice image and the segmentation results.

**Figure 9:**
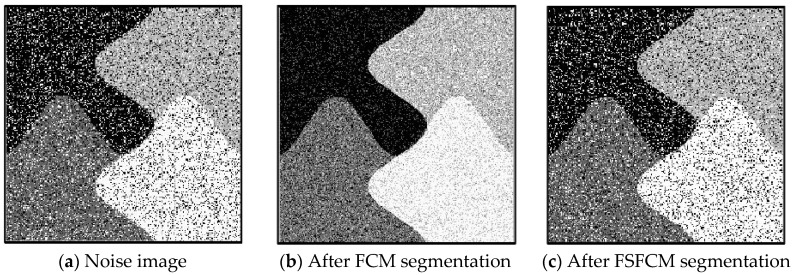

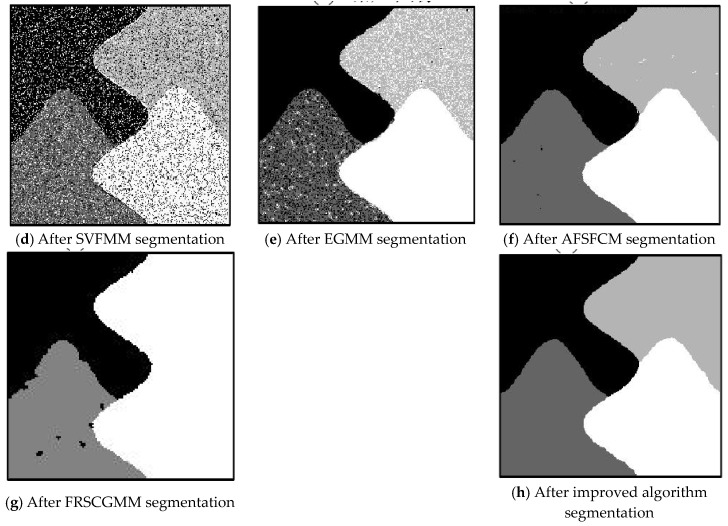
Salt-and-pepper noise interfering with the image of four man-made structures and the segmentation results.

**Figure 10:**
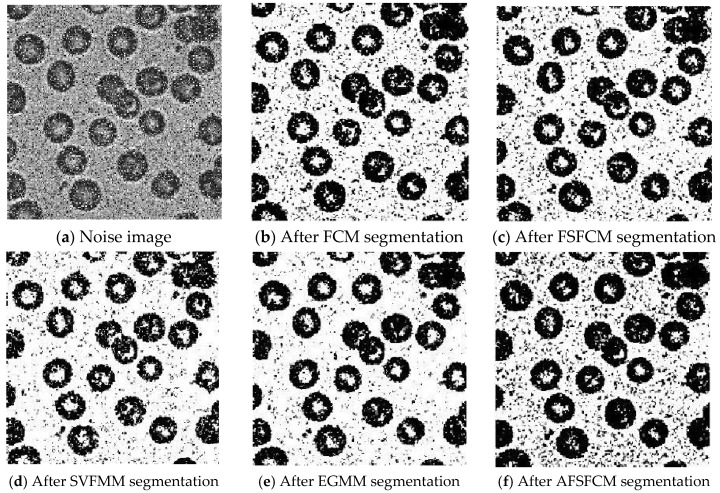

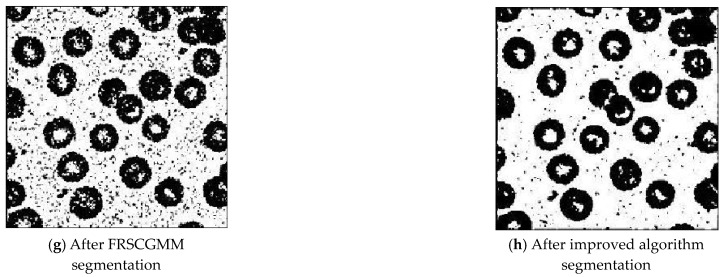
Salt-and-pepper noise interfering with the image of a cell and the segmentation results.

**Figure 11:**
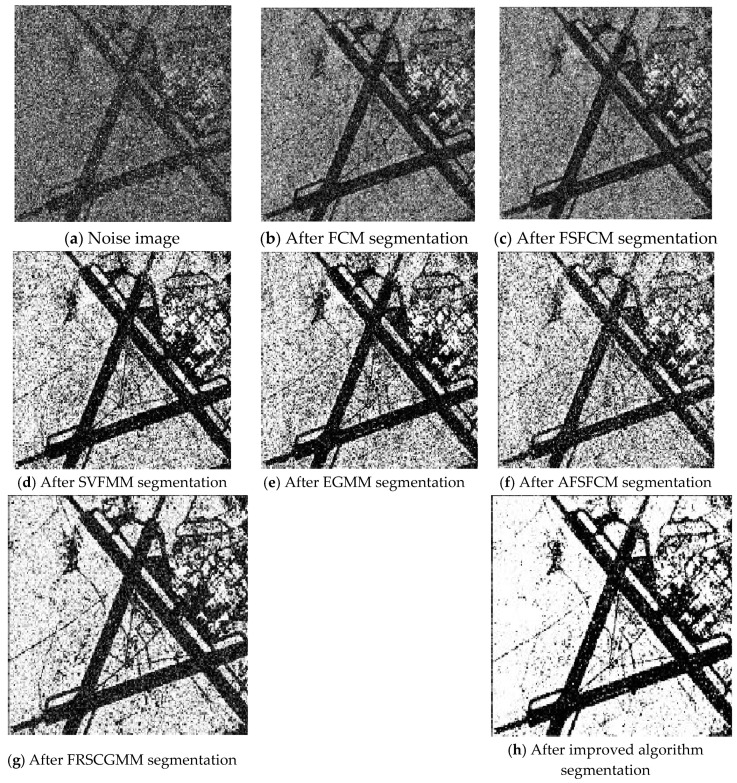
Salt-and-pepper noise interfering with remote sensing image 6 and the segmentation results.

Salt-and-pepper noise (35%) is added to the Lena image and the four synthetic greyscale images, and a higher level of salt-and-pepper noise (45%) is added to the CT slices of the brain tissue. Moreover, 40% salt-and-pepper noise is added to the cell image and remote sensing image 6. The results of the segmentation test are shown in Figure 7, Figure 8, Figure 9, Figure 10 and Figure 11. The PSNR and error rate of the segmentation results are shown in Table 3 and Table 4.

From the anti-salt-and-pepper noise segmentation results in Figure 7, Figure 8, Figure 9, Figure 10 and Figure 11, it can be observed that the segmentation results for the FCM, FSFCM and SVFMM algorithms are seriously polluted. The results of the AFSFCM algorithm proposed in this paper contain less noise than those of the EGMM. The FRCSGMM algorithm can separate the background from the segmented target, but compared with the segmentation result of the improved algorithm, the FRCSGMM result still contains some noise. As shown in Figure 8, the results of the four artificial image segmentation algorithms indicate that the FRCSGMM algorithm results in some misclassified regions in the results and cannot correctly classify the number of categories. However, the improved algorithm yields complete segmentation results and almost no noise, and it largely restores the original image information. Combined with the PSNR in Table 3, it can be concluded that the AFSFCM algorithm has improved resistance to salt-and-pepper noise compared to the EGMM. Compared with the FCM, FSFCM, SVFMM, AFSFCM and EGMM algorithms, the improved method can better suppress salt-and-pepper noise interference. Table 4 shows that the improved algorithm has the lowest error rate, followed by the FRCSGMM algorithm. The PSNR of the improved algorithm is 1.5501–13.4396 dB higher than that of the other algorithms, and the MSC is 5.02–41.05% lower than that of the other algorithms. This finding verifies that the improved algorithm has the best segmentation performance.

### 3.3. Image Segmentation Test with Multiplicative Noise

**Figure 12:**
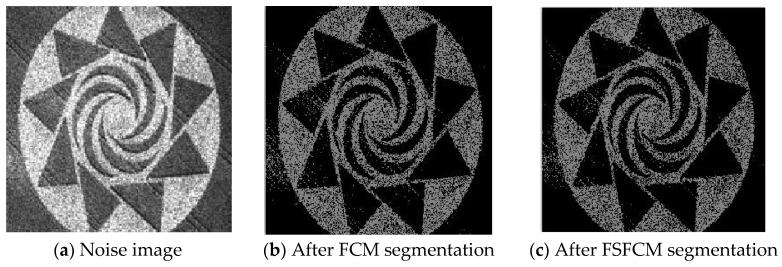

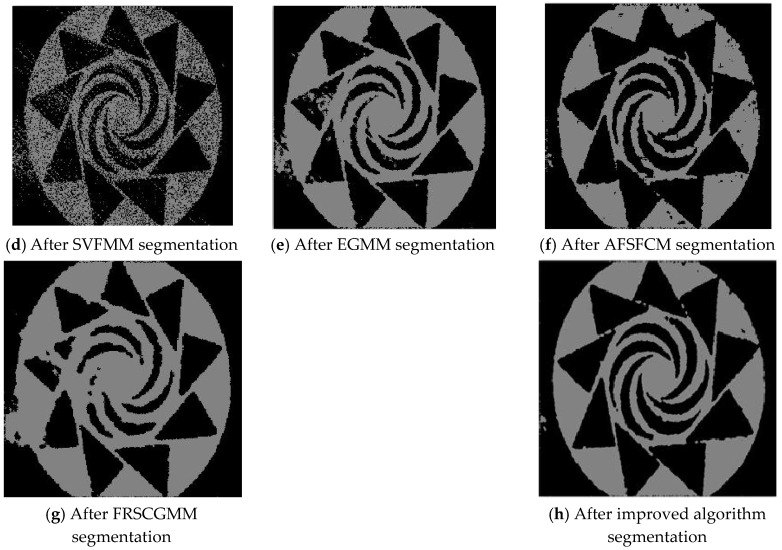
Multiplicative noise interfering with the wheat field image and the segmentation results.

**Figure 13:**
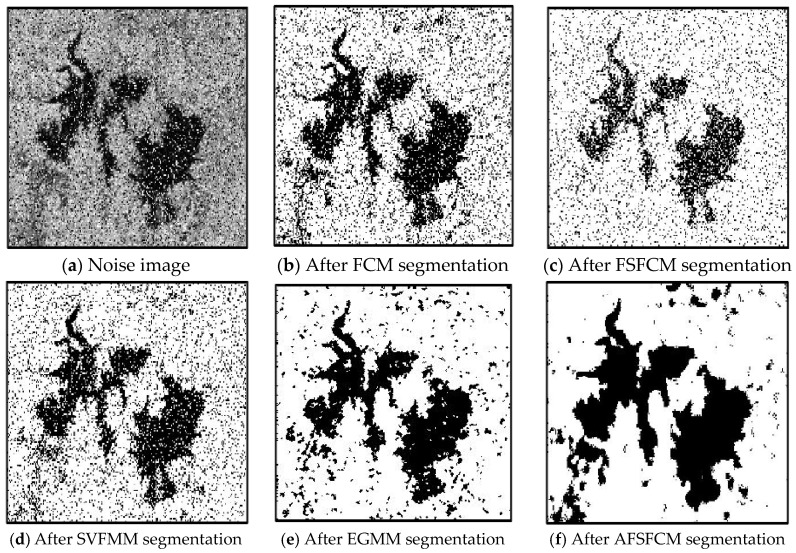

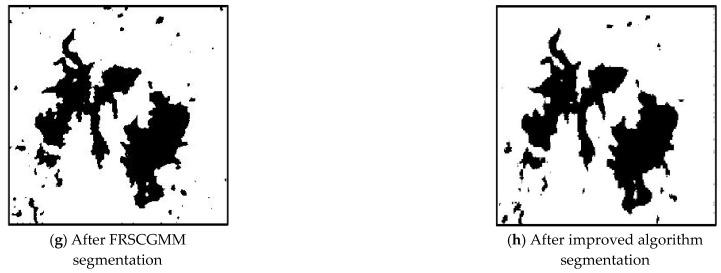
Multiplicative noise interfering with the canyon image and the segmentation results.

**Figure 14:**
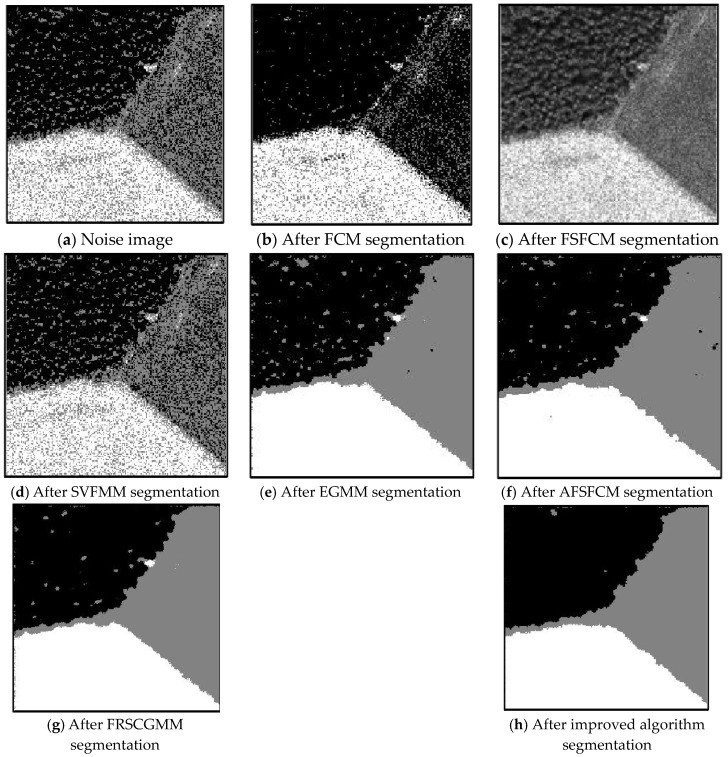
Multiplicative noise interfering with the forest image and the segmentation results.

**Figure 15:**
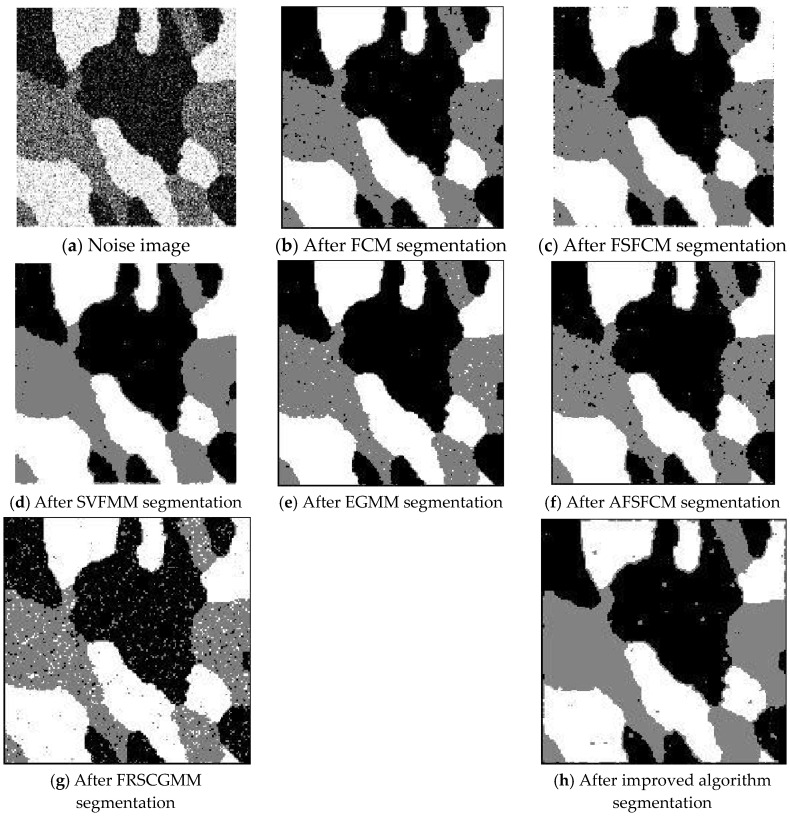
Mixed noise interfering with the composite image (1) and the segmentation results.

The remote sensing images of wheat fields, canyons and forests had multiplicative noise added with a mean value of 0 and mean squared deviations of 90, 121 and 61, respectively [50,51,52]. The numbers of clusters were 2, 2 and 3, respectively. Multiplicative noise with a mean value of 0 and a mean square deviation of 70 was added to the synthetic image, and the number of clusters was 2. The results are shown in Figure 12, Figure 13, Figure 14 and Figure 15 and Table 5 and Table 6.

According to the results of remote sensing image segmentation in Figure 12, Figure 13, Figure 14 and Figure 15, compared with the improved algorithm, the FCM, FSFCM and SVFMM algorithms yield results that contain considerable noise. Compared with the improved algorithm, the EGMM and FRSCGMM algorithms can basically distinguish between forest, farmland and bare land; however, there are still some grasslands in the forest area, resulting in the incomplete segmentation of the forest area. Furthermore, the segmentation result of the improved algorithm is a more complete segmentation according to the type of feature, and the edges are more continuous than those of the other methods. In the FRSCGMM algorithm, the shape of the wheat field is incomplete. The AFSFCM algorithm can divide the wheat field completely. Compared with in the result of the improved algorithm, the two types of wheat fields in the AFSFCM result are more completely divided, and most of the noise is suppressed. According to the results of synthetic image segmentation, there is considerable noise with the other algorithms. In this paper, the algorithm can reduce noise well and segment the images completely and continuously. Based on Table 5 and Table 6, the PSNR of the improved algorithm is 1.9113–11.2613 dB higher than that of the other algorithms, and the MSC is 0.3–21.79% lower than that of the other algorithms. In summary, the improved algorithm has a stronger ability to resist multiplicative noise than the other methods, and the segmentation performance is better.

### 3.4. Image Segmentation Test with Mixed Noise

**Figure 16:**
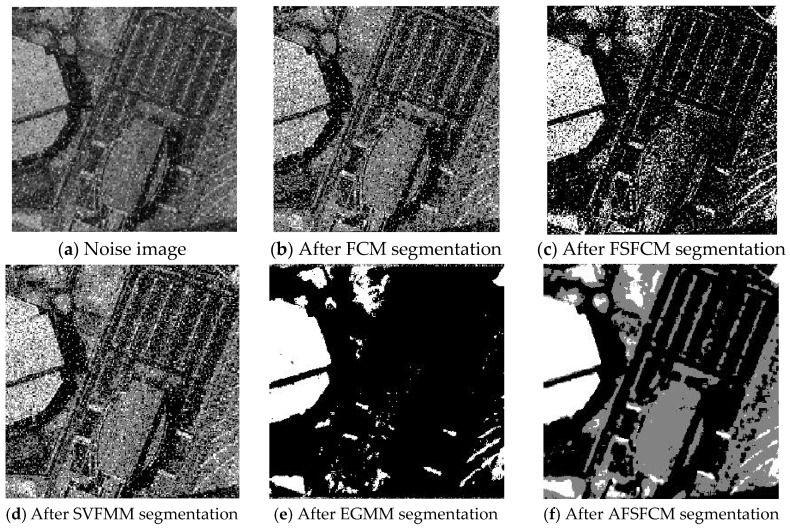

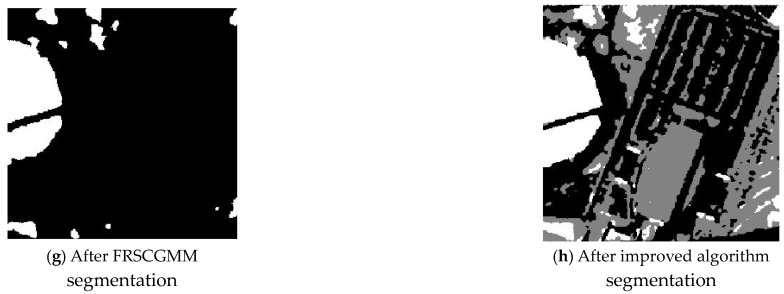
Mixed noise interfering with the house image and the segmentation results.

**Figure 17:**
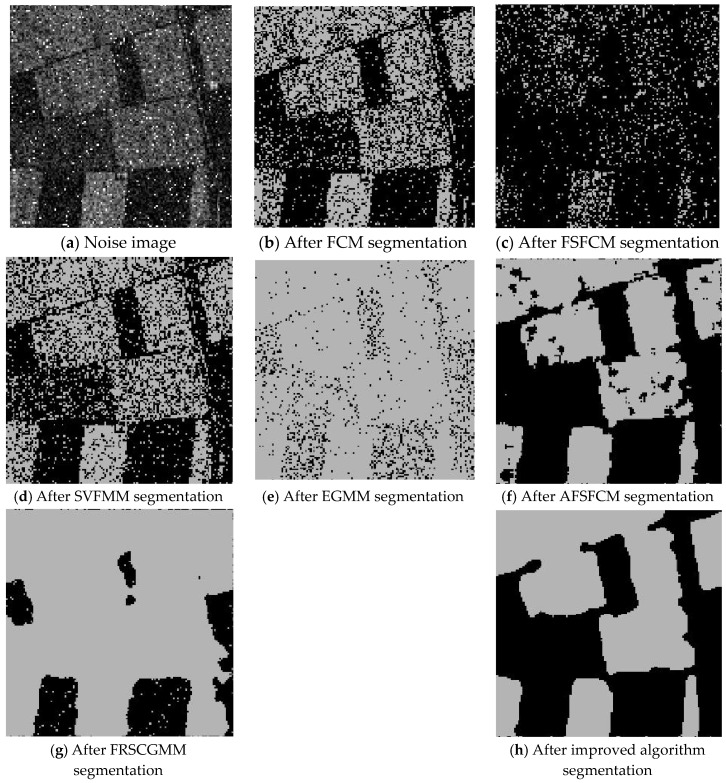
Mixed noise interfering with the farmland image and the segmentation results.

**Figure 18:**
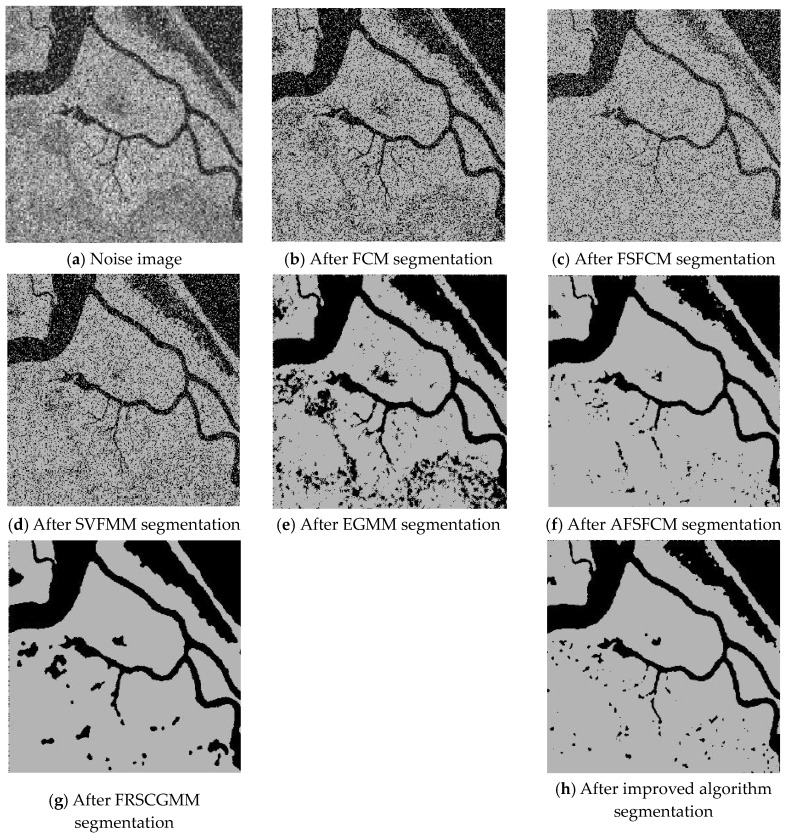
Mixed noise interfering with the river image and segmentation results.

**Figure 19:**
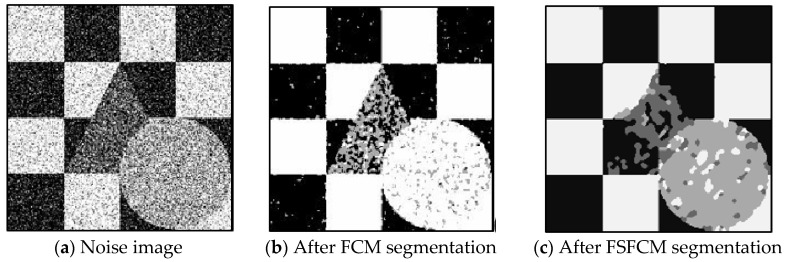

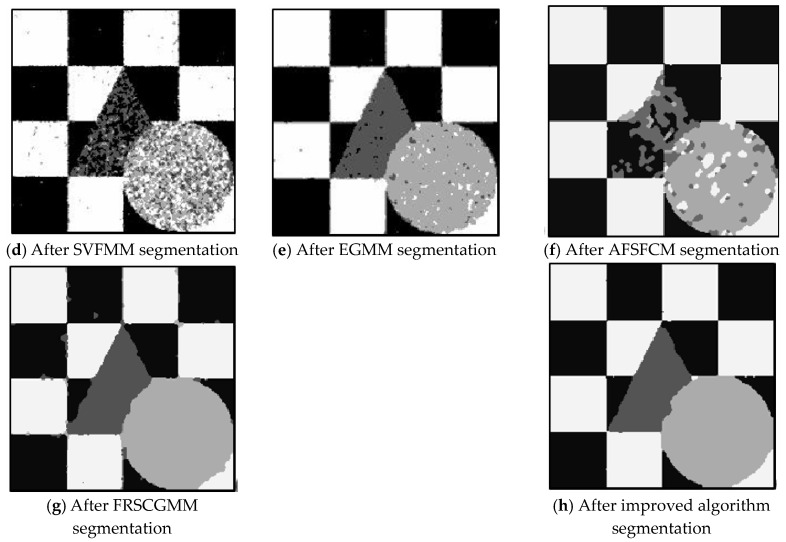
Mixed noise interfering with composite image (2) and the segmentation results.

**Figure 20:**
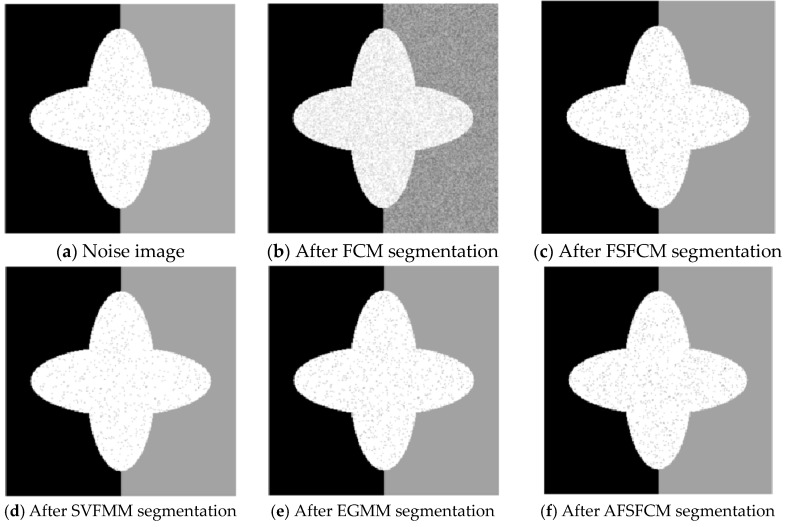

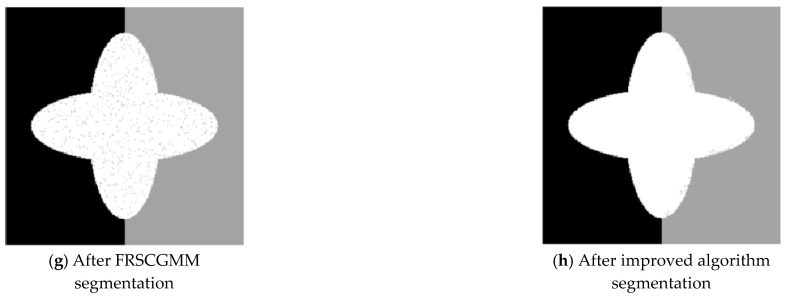
Mixed noise interfering with composite image (3) and the segmentation results.

Three remote sensing images, including a stadium, farmland and rivers, had Gaussian noise added (mean value of 0 and mean squared error of 25) along with different intensities of salt-and-pepper noise (6%, 15% and 35%). The images were obtained from a segmentation test. The numbers of clusters were 2, 3 and 2. Gaussian noise with a mean value of 0 and a mean square error of 30%, 20% and 30% and salt-and-pepper noise were added to synthetic images 2 and 3, with 2 clusters each [53,54]. The segmentation results are shown in Figure 16, Figure 17, Figure 18, Figure 19 and Figure 20.

The segmentation test results for the remote sensing images shown in Figure 16, Figure 17, Figure 18, Figure 19 and Figure 20 indicate that the FCM and FFSCM algorithms have large numbers of noise points. The SVFMM algorithm considers the influence of the neighbourhood space, and it suppresses noise points. However, the ability of this algorithm is limited, and there are still many noise points in the segmentation results. From the farmland segmentation results in Figure 17, we see that the EGMM and FRSCGMM algorithm segmentations produce different degrees of misclassification, and these methods cannot identify the location of the farmland. In the stadium segmentation results in Figure 18, the EGMM and FRCSGMM algorithm results lack information for the stadiums, runways, houses and green spaces, and sample information cannot be effectively extracted from the remote sensing images [55]. The AFSFCM algorithm basically recognises and extracts the stadium information, but it yields more noise points than the improved algorithm. Regarding the results of the river segmentation test in Figure 18, the AFSFCM algorithm segmentation results contain less noise than the other results, and the improved algorithm segmentation results are almost noiseless. For artificial synthetic images 2 and 3, most of the algorithms have much noise, and the improved algorithm has the least noise. The PSNR and the misclassification values for the remote sensing image segmentation results are shown in Table 7 and Table 8. The PSNR of the improved algorithm is 1.0233–10.2804 dB higher than that of the other algorithms, and the MSC is 0.9–30.95% lower than that of the other algorithms. Compared with the other five algorithms, the improved algorithm is more suitable for image segmentation when salt-and-pepper noise and Gaussian mixed noise interference exist.

### 3.5. The Limitations of the Algorithm Proposed in this Paper and Its Research Prospects

In this paper, the fuzzy C-means clustering algorithm is studied and improved, and feature selection and Markov constraint algorithms are added to enhance the anti-noise robustness of the overall algorithm. Although the anti-noise performance and segmentation performance of the algorithm proposed in this paper are improved compared to those of traditional methods, the image segmentation algorithm needs to be applied in different fields. Therefore, the algorithm proposed in this paper still has some problems. Here are some of the key issues to be considered in the future:

1. This paper proposes a robust clustering algorithm for image segmentation with embedded neighbourhood pixels and proposes a robust clustering method for greyscale images. This paper only tests greyscale images, but there are many factors that need to be considered when segmenting colour images. Therefore, further improving the segmentation performance for colour images needs to be further studied. The next research direction will be improving the segmentation performance for colour images.
2. The robust clustering segmentation algorithm based on feature selection studied in this paper adds neighbourhood information constraints, and one of the difficulties with the algorithm is selecting regular coefficients and fuzzy coefficients. In this paper, the algorithm for the manual selection of parameters needs to be debugged. Further research is needed regarding the adaption of the algorithm.
3. Because of the uncertainty and differences among images, improving the universality and adaptability of the algorithm needs to be further studied. In image segmentation, the algorithm proposed in this paper sets the corresponding number of clusters based on the specific image to be segmented, and an automatic method of determining the number of clusters is lacking. The selection of the number of clusters will directly affect the result of image segmentation. Therefore, selecting the appropriate number of clusters adaptively should be considered in follow-up studies.

## 4. Conclusions

To overcome the insufficient robustness of the GMM clustering algorithm for feature selection, we propose a feature selection embedded Gaussian mixture algorithm in the neighbourhood space that considers the correlation of the pixel neighbourhood space and the Markov random space. Moreover, the a priori probability is defined. This approach is combined with the FSGMM and embedded in the regular fuzzy clustering algorithm with KL divergence to improve the noise resistance and robustness of the algorithm. In addition, the results obtained by using traditional FCM clustering to segment images are used as the initialisation parameters for the improved algorithm, and it is found that the algorithm should be avoided since it is limited to the local optimal solution and cannot obtain a good segmentation effect. Gaussian noise, salt-and-pepper noise, multiplicative noise and mixed noise are added to different types of images, and after comparing the anti-noise PSNRs and MCRs of various algorithms, it is verified that the improved algorithm yields good segmentation results with high consistency and anti-noise robustness. The proposed method is effective and meets the needs of noisy image segmentation. In future work, we plan to improve the algorithm in the following three aspects: 1. improve the segmentation performance for colour images; 2. improve the self-adaptability of the algorithm and select regular and fuzzy coefficients; and 3. select the appropriate number of clusters adaptively.

## Figures and Tables

**Table 1:** Comparison of the anti-Gaussian noise peak signal-to-noise ratio (PSNR) (dB) of each algorithm.

Split Image	FCM	FSFCM	SVFMM	EGMM	AFSFCM	FRSCGMM	Improved Algorithm
Lena image	10.973	9.3729	11.1786	13.5664	15.0928	15.8741	16.0013
Four types of man-made objects	12.4832	11.6124	14.8312	15.8812	18.2512	24.2871	25.5661
Cameraman	9.8102	11.4171	9.8532	14.7561	15.2704	15.8812	16.4271
Brain slice	8.0573	12.6513	10.0012	12.6712	12.7812	13.9812	13.5148
Remote sensing image 7	14.2369	15.7231	17.2645	18.2651	19.8432	25.2813	27.2172

**Table 2:** Comparison of the anti-Gaussian noise misclassification rate (MCR) (%) of each algorithm.

Split Image	FCM	FSFCM	SVFMM	EGMM	AFSFCM	FRSCGMM	Improved Algorithm
Lena image	41.31	44.01	36.39	21.13	14.1	12.31	10.12
Four types of man-made objects	33.28	34.34	26.69	18.53	16.98	2.1	1.78
Cameraman	26.15	11.76	16.12	3.24	2.79	3.01	1.96
Brain slice	13.13	9.23	11.59	5.65	4.45	4.67	3.87
Remote sensing image 7	39.28	34.23	26.98	19.54	17.28	3.12	1.96

**Table 3:** Comparison of the PSNRs (dB) of various algorithms with salt-and-pepper noise.

Split Image	FCM	FSFCM	SVFMM	EGMM	AFSFCM	FRSCGMM	Improved Algorithm
Lena image	7.5615	11.2314	8.1516	8.9134	10.0712	10.3151	12.9512
Brain slice	7.0812	7.1212	6.4134	13.9215	14.0021	15.7121	17.2612
Four types of man-made objects	9.3125	9.1541	9.3123	16.4312	21.0141	17.2571	22.7521
Cell	10.2319	11.2589	9.2417	11.2913	12.2876	17.2871	21.7545
Remote sensing 6	12.2786	13.2684	14.1215	14.2651	17.2654	18.2561	23.7612

**Table 4:** Comparison of the anti-salt-and-pepper noise MCR (%) of each algorithm.

Split Image	FCM	FSFCM	SVFMM	EGMM	AFSFCM	FRSCGMM	Improved Algorithm
Lena image	36.81	40.81	36.53	45.12	17.41	14.19	13.17
Brain slice	41.13	41.24	23.01	13.56	12.15	11.14	6.12
Four types of man-made objects	36.12	39.61	22.19	14.21	13.28	24.61	2.51
Cell	39.28	30.21	23.38	17.36	15.67	11.26	3.14
Remote sensing 6	45.28	40.21	38.12	25.23	16.19	12.15	4.23

**Table 5:** Comparison of the anti-multiplier noise PSNR (dB) of each algorithm.

Split Image	FCM	FSFCM	SVFMM	EGMM	AFSFCM	FRSCGMM	Improved Algorithm
Wheat field	14.1793	13.8612	14.2325	18.4912	18.5945	17.1254	22.4898
Canyon	8.1982	8.3235	8.4715	11.2412	11.3671	13.3761	15.2874
Forest	12.2781	10.6421	12.4312	20.7131	22.1765	21.4981	23.5412
Composite image (1)	6.2341	6.4987	7.2736	8.1652	9.2541	11.5432	13.7645

**Table 6:** Comparison of the anti-multiplicative noise MCRs (%) of various algorithms.

Split Image	FCM	FSFCM	SVFMM	EGMM	AFSFCM	FRSCGMM	Improved Algorithm
Wheat field	14.81	15.61	14.51	5.51	5.21	7.58	2.18
Canyon	15.92	15.13	14.41	5.41	5.21	7.61	2.58
Forest	23.91	32.51	22.81	3.39	2.42	2.92	2.12
Composite image (1)	11.26	10.98	9.65	4.21	4.08	3.12	1.45

**Table 7:** Comparison of the anti-mixed noise PSNR (dB) of each algorithm.

Split Image	FCM	FSFCM	SVFMM	EGMM	AFSFCM	FRSCGMM	Improved Algorithm
Stadium	9.3761	8.4231	9.6312	9.1671	14.2512	8.3851	15.4321
Farmland	8.8629	6.4431	9.2451	7.4512	14.8725	8.8136	16.7235
River	11.0791	11.0568	10.5571	12.4812	16.7891	14.7812	18.5821
Composite image (2)	7.2165	8.1678	9.2672	10.2640	13.2836	6.1234	14.2123
Composite image (3)	6.2123	7.4256	8.2367	10.2341	12.2123	5.2121	13.2356

**Table 8:** Comparison of the anti-mixed noise MCR (%) of each algorithm.

Split Image	FCM	FSFCM	SVFMM	EGMM	AFSFCM	FRSCGMM	Improved Algorithm
Stadium	35.12	45.87	34.91	42.16	14.21	42.65	11.21
Farmland	25.31	46.51	24.12	38.12	6.81	27.21	5.21
River	19.71	20.12	19.12	12.76	4.13	6.81	3.61
Composite image (2)	16.21	17.54	14.58	11.26	3.21	4.87	2.31
Composite image (3)	17.74	18.21	15.54	13.23	4.76	5.76	3.21
